# Long-Term Management After Variceal Bleed — The Current Role of Sclerotherapy

**DOI:** 10.1155/1991/74906

**Published:** 1991

**Authors:** P. C. Bornman, J. E. J. Krige, J. P. Dunn, J. Terblanche

**Affiliations:** Surgical Gastroenterology E23, Groote Schuur Hospital and the Medical Research Council Liver Research Centre Department of Surgery University of Cape Town Cape Town Observatory 7925 South Africa

## Abstract

While injection sclerotherapy has been accepted as the treatment of choice for acute variceal bleeding, its role as a definitive long-term treatment modality has not yet been clearly defined. This paper will critically analyse the current status of this technique, now widely used, and a comparison will be made with conventional medical management. The review will be based on the 10 years' Cape Town experience and the published series on this subject. A long-term management strategy will also be discussed.


*HPB Surgery*, 1991. Vol. 4, pp 5–10
Reprints available directly from the publisher
Photocopying permitted by license only

© 1991 Harwood Academic Publishers GmbH
Printed in the United Kingdom

### LONG-TERM MANAGEMENT AFTER VARICEAL BLEED — THE CURRENT ROLE OF SCLEROTHERAPY

*P.C. BORNMAN, J.E.J. KRIGE, J.P. DUNN and J. TERBLANCHE
*From Surgical Gastroenterology, Groote Schuur Hospital and the Medical Research Council, Liver Research Centre, Department of Surgery, University of Cape Town, South Africa.*

*(Received 21 September 1990)*

While injection sclerotherapy has been accepted as the treatment of choice for acute variceal bleeding, its role as a definitive long-term treatment modality has not yet been clearly defined. This paper will critically analyse the current status of this technique, now widely used, and a comparison will be made with conventional medical management. The review will be based on the 10 years' Cape Town experience and the published series on this subject. A long-term management strategy will also be discussed.

KEY WORDS: Sclerotherapy, eosophageal varices

### INTRODUCTION

Dissatisfaction with the results of management of bleeding oesophageal varices at Groote Schuur Hospital in the seventies[1,2] prompted the Liver Research Group in Cape Town to launch prospective studies to evaluate the role of sclerotherapy both during the acute bleeding episode[3] and as an alternative definitive form of long-term treatment[4].

### LONG-TERM SCLEROTHERAPY VERSUS MEDICAL TREATMENT

In the first long-term controlled study, repeated sclerotherapy (with the aim of eradication of varices) was compared to a medical management regimen. Emergency sclerotherapy was however instituted when balloon tamponade was required to control recurrent bleeding episodes. Although recurrent acute variceal bleeds were appreciably reduced by sclerotherapy, and eradication of varices was usually possible, overall mortality was not reduced[4,5].

Failure to improve survival was also reported in two further studies[6,7] where the control groups did not receive emergency salvage sclerotherapy for recurrent

Correspondence to: *Professor P.C. Bornman, Surgical Gastroenterology E23, Groote Schuur Hospital, Observatory 7925, Cape Town, South Africa.

variceal bleeds. However, in Soderland's[6] and Korula's[7] studies, fewer deaths from recurrent variceal bleeding occurred in patients who received sclerotherapy. Improved survival was also calculated in the Los Angeles Study[7] when patients receiving shunt surgery were excluded from the survival analysis. The Kings College[8] and Copenhagen[9] trials also showed a significantly improved long-term survival associated with fewer recurrent variceal bleeds in the sclerotherapy group. The difference in the latter study was, however, only evident 40 days after the index bleed.

The differences in the various trials comparing sclerotherapy with medical treatment have highlighted the difficulties in interpretation of treatment regimens in such a complicated condition. There are many variables which could influence the outcome including timing of therapy, entry criteria, aetiology and severity of the underlying liver disease, management strategies, techniques of sclerotherapy and continued alcohol abuse. The failure to show benefit with injection sclerotherapy in the Cape Town study could be explained by the use of salvage emergency sclerotherapy in the medically treated control group[4,5]. In the Kings College study the entry of patients only after they had survived the initial period might have introduced a bias[8]. Nevertheless, the Copenhagen study did show a clear long-term benefit of sclerotherapy in terms of reduced recurrent variceal bleeding and related mortality[9].

On balance, current knowledge based on the results of controlled and uncontrolled studies would suggest that patients in whom varices are successfully eradicated fare better than those who receive conventional medical therapy.

### CAPE TOWN 10 YEARS' EXPERIENCE

A recent review of our total experience in the long-term management of 245 patients who have bled from varices has again confirmed sclerotherapy as a viable definitive form of treatment for the majority of patients[10]. Alcoholic cirrhosis was the commonest aetiology (57%) and the Pugh-Childs risk gradings were A: 72 (40 portal vein thrombosis), B: 81, C: 78, unknown: 14. Flexible fibreoptic endoscopy under sedation replaced rigid oesophagoscopy half-way through the study period. A combined intra and paravariceal injection technique was employed using ethanolamine oleate as sclerosant.

This study has confirmed both our own[4,5] and others[6–9] previous finding that varices can be eradicated in the majority of patients, but showed that this required a median of five injection sclerotherapy sessions over a mean period of 9.25 months. Nevertheless the prognosis in patients in whom varices were eradicated was good and the risk of rebleeding low (10.6%).

There remains, however, a hard core of patients who develop recurrent variceal bleeds after their first admission. In this series 41 patients (17%) had no less than 119 variceal bleeding episodes during 81 subsequent admissions, and 53 per cent of the episodes required the addition of balloon tamponade for control. While dealth was directly attributed to bleeding in only 8 patients, it is conceivable that some of the "liver failure" deaths could have been due to massive blood loss and transfusion.

Although the complications of sclerotherapy were cumulative with time, only few were life-threatening. There were 17 (7%) injection site leaks and all settled on conservative treatment. However this resulted in treatment delays and some deaths were due to recurrent bleeding and liver failure during such delays. Four oesophageal ruptures after rigid oesophagoscopy were more serious and were associated with two deaths. Most of the 52 per cent mortality of the 245 patients over the 10 year period was due to liver failure. The prognosis was predictably poor in Childs C alcoholic cirrhotic patients but a continued analysis of the results of sclerotherapy in patients with extrahepatic portal vein thrombosis confirmed the excellent results of a previous communication from our unit[11] and from others[12].

### TECHNIQUE

The rigid oesophagoscope, which ushered in the new era of enthusiasm for sclerotherapy[5,13,14] in the seventies, has now been superseded by the flexible fibreoptic endoscope[15]. It has proved to be as effective as the rigid scope in one controlled study [16] both in acute and long-term management. It is safer than the rigid scope, does not require a general anaesthetic and most patients can be treated more cost-effectively on an outpatient basis. Needless to say this is a major cost-saving factor. Earlier reports advocated the use of an outer sheath[17] or some form of balloon compression[18], but simpler free-hand injection has become the most widely used technique. However, the use of an outer sheath does seem to speed-up eradication[17,19] and should be considered in difficult cases. The novel endoscopic ligation device[20] seems promising and the results of controlled studies comparing this with sclerotherapy are awaited with interest.

While most workers agree that the lower oesophagus is the most important site for sclerosing varices, it remains uncertain whether the injection should be intravariceal. To produce thrombosis of the varix, or paravariceal in the submucosal plane to evoke mucosal and submucosal thickening. The intravariceal technique has been used widely in Great Britain and the United States while most European workers continue to use the submucosal technique. So far only one prospective randomised trial has addressed this problem, in which the intravariceal technique proved superior[21]. We continue to use a combined intra- and paravariceal technique, the latter mostly during the initial sclerotherapy sessions to prevent troublesome needle puncture bleeds from large variceal channels.

Kitano *et al.*[22] recently proposed a modified two stage intravariceal and paravariceal technique making use of an overtube. Thrombosis of the varices is first induced by intravariceal injections, followed by the deliberate promoting of mucosal ulceration with submucosal injections. This technique has markedly reduced the incidence of recurrent varices and bleeding in their patients.

The best choice of sclerosant also remains uncertain. The most widely used agents are ethanolamine oleate and sodium tetradecyl sulfate for intravariceal injection and polidocanol for paravariceal injections. Again there is a paucity of controlled trials evaluating the efficacy of these agents. Two studies by one group have shown ethanolamine oleate to be superior to polidocanol[23] and to sodium tetradecyl sulfate[24] while polidocanol was more effective than absolute alcohol when investigated by another group[25]. On balance it would appear that there is no clear superior sclerotherapy technique or sclerosing agent when these procedures are carried out by experts.

Since eradication of oesophageal varices is associated with a lower incidence of recurrent variceal bleeding, it would seem important to achieve this goal in the shortest possible time. Three controlled studies have shown that a one week interval treatment schedule achieves earlier variceal obliteration than when treatments are spaced by two[26] or three[27,28] weeks. The incidence of rebleeding, however, was only significantly reduced in one study[28] and in none of the studies was there a reduction in mortality. A higher incidence of oesophageal ulceration in the "one week" regimen delayed treatment in a considerable number of patients and may explain why this regimen was not unequivocally better than the "three week" one.

### CONCLUSIONS

Repeated sclerotherapy by virtue of its simplicity and safety, has earned a rightful place in the long-term treatment of patients after variceal bleeding and has become the treatment of choice in Cape Town. It would now seem to be the preferred treatment for Child C and most Child B patients who tolerate surgical intervention poorly. Sclerotherapy does not impair liver function or promote hepatic encephalopathy and therefore makes it a strong contender in the treatment of good risk patients as well. In patients with extrahepatic portal vein thrombosis, sclerotherapy has proved to be as good as or even better than devascularization operations.

Recurrent variceal bleeding remains the most limiting aspect of long-term sclerotherapy. While the risk diminishes with time as the variceal channels are obliterated, some recurrent bleeds are fatal or contribute to deaths from liver failure. Defining sclerotherapy failures remains a problem. The number of sclerotherapy sessions and the time taken to eradicate varices are not necessarily measures of success. Many of the patients who require more than the average number of sclerotherapy sessions ultimately do well and even patients with persistent varices may not bleed again and succumb from causes other than recurrent variceal bleeding. We believe that patients who develop life-threatening variceal bleeding after an adequate course of treatment should be regarded as failures of long-term treatment and in these an early salvage operation as proposed by Warren *et al*[29] and Cello *et al.*[30] would seem to give the best results.

Our current long-term management policy is to submit patients to a weekly sclerotherapy session with the aim of early eradication, appreciating that factors such as the development of injection site ulceration and patient compliance frequently interfere with the sclerotherapy programme. After eradication of the varices patients should be followed up at six to 12 month intervals and, if varices recur, an aggressive sclerotherapy schedule should be instituted again. It is hoped that in the future the addition of effective pharmacological agents will improve the results of sclerotherapy both in terms of facilitating earlier eradication and prevention of recurrence.

### *References*

1. Terblanche, J., Saunders, S.J., Louw, J.H. (1974) Cirrhosis of the liver: Acute hepatic failure. In: *Surgical Forum — The Liver*. Ed. Smith, R., pp. 1–35 London, Butterworth
2. Novis, B.H., Duys, P., Barbezat, G.O., Clain, J., Bank, S. and Terblanche, J. (1976) Fibreoptic endoscopy and the use of the Sengstaken tube in acute gastrointestinal haemorrhage in patients with portal hypertension and varices. *Gut*, **17** 258–263

3. Terblanche, J., Northover, J.M.A., Bornman, P.C., Kahn, D., Barbezat, G.O., Sellars, S.L. and Saunders, S.J. (1979) A prospective evaluation of injection sclerotherapy in the treatment of acute bleeding from esophageal varices. *Surgery*, **85** 239–245
4. Terblanche, J., Northover, J.M.A., Bornman, P.C., Kahn, D., Silber, W., Barbezat, G.O., Sellars, S., Campbell, J.A.H. and Saunders, S.J. (1979) A prospective controlled trial of sclerotherapy in the long-term management of patients after esophageal variceal bleeding. *Surgery Gynecology Obstetrics*, **148** 323–333
5. Terblanche, J., Bornman, P.C., Kahn, D., Jonker, M.A.T., Campbell, J.A.H., Wright, J.P. and Kirsch, R. (1983) Failure of repeated injection sclerotherapy to improve long-term survival after oesophageal variceal bleeding. A five year prospective controlled clinical trial. *Lancet*, **2** 1328–1332
6. Soderland, C. and Ihre, T. (1985) Endoscopic sclerotherapy vs conservative management of bleeding oesophageal varices. A 5 year prospective controlled trial of emergency and long-term treatment. *Acta Chirurgica Scandavica* **151** 449-456
7. Korula, J., Balart, L.A., Radvan, G., Zweiban, B.E., Larson, A.W., Kao, H.W. and Yamada, S. (1985) A prospective randomised controlled trial of chronic esophageal variceal sclerotherapy. *Hepatology*, **5** 584–589
8. Westaby, D., MacDougall, B.R.D. and Williams, R. (1985) Improved survival following injection sclerotherapy for esophageal varices: Final analysis of a controlled trial. *Hepatology*, **5** 827–830
9. The Copenhagen esophageal varices sclerotherapy project (1984) Sclerotherapy after first variceal haemorrhage in cirrhosis: A randomised multicenter trial. *New England Journal of Medicine,* **311** 1594–1600
10. Terblanche, J., Kahn, D. and Bornman, P.C. (1989) Long-term injection sclerotherapy treatment for esophageal varices – A 10 year prospective evaluation. *Annals of Surgery*, **210** 725–731
11. Kahn, D., Terblanche, J., Kitano, S. and Bornman, P.C. (1987) Injection sclerotherapy in adult patients with extrahepatic portal vein thrombosis. *British Journal of Surgery*, **74** 600–602
12. Chawla, Y.K., Dilawari, J.B., Ramesh, G.N., Kaur, U., Mitra, S.K. and Walia, B.N.S. (1990) Sclerotherapy in extrahepatic portal venous obstruction. *Gut*, **31** 213–216
13. Johnston, G.W. and Rodgers, H.W. (1973) A review of 15 years experience in the use of sclerotherapy in the control of acute haemorrhage from oesophageal varices. *British Journal of Surgery* **60** 797–800
14. Paquet, K-J. and Oberhammer, E. (1978) Sclerotherapy of bleeding oesophageal varices by means of endoscopy. *Endoscopy*, **10** 7–12
15. Terblanche, J., Burroughs, A.K. and Hobbs, K.E.F. (1989) Controversies in the management of bleeding esophageal varices. *New England Journal of Medicine*, **320** 1469–1475
16. Bornman, P.C., Kahn, D., Terblanche, J., Worthley, C., Spence, R.A.J. and Krige, J.E.J. (1988) Rigid versus fibreoptic endoscopic injection sclerotherapy. *Annals of Surgery*, **208** 175–178
17. Westaby, D., MacDougall, B.R.D. and Melia, W. (1983) A prospective randomised study of two sclerotherapy techniques for oesophageal varices. *Hepatology*, **3** 681–684
18. Lewis, J., Chung, R.S. and Allison, J. (1980) Sclerotheraphy of esophageal varices. *Archives of Surgery*, **115** 476–480
19. Kitano, S.N., Koyanagi, Y.I., Iso, Y., Iwanaga T., Higashi, H. and Sugimachi, K. (1987) Prospective randomised trial comparing two injection techniques for sclerosing oesophageal varices: over-tube and free-hand. *British Journal of Surgery*, **74** 603–606
20. Stiegmann, G.V., Goff, J.S., Sun, J.H., Davis, D. and Bozdech, J. (1989) Endoscopic variceal ligation: an alternative to sclerotherapy. *Gastrointestinal Endoscopy*, **35** 431–434
21. Sarin, S.K., Nanda, R., Sachdev, G., Chari, S., Anand, B.S. and Broor, S.L. (1987) Intravariceal versus paravariceal sclerotherapy: a prospective controlled randomised trial. *Gut*, **28** 657–662
22. Kitano, S., Koyanagi, N., Iso, Y., Higashi, H. and Sugimachi, K. (1987) Prevention of recurrence of esophageal varices after endoscopic injection sclerotherapy with ethanolamine oleate. *Hepatology*, 7 810–815
23. Kitano, S., Iso, Y., Koyanagi, N., Higashi, H. and Sugimachi, K. (1987) Ethanolamine oleate is superior to polidocanol (Aethoxysklerol) for endoscopic injection sclerotherapy of esophageal varices: a prospective randomized trial. *Hepatogastroenterology*, **34** 19–23
24. Kitano, S., Iso, Y., Yamaga, H., Hashizume, M, Higashi, H. and Sugimachi, K. (1988) Trial of sclerosing agents in patients with oesophageal varices. *British Journal of Surgery*, **75** 751–753
25. Atamkuri, S.P., Bhargava, D.K. and Sharma, M.P. (1988) Endoscopic sclerotherapy for esophageal varices: A prospective, randomised trial of absolute alcohol versus polidocanol. *Indian Journal of Gastroenterology*, 7 87–89

26. Higashi, H., Kitano, S., Hashizume, M., Yamaha, H. and Sugimachi, K. (1989) A prospective randomized trial of schedules for sclerosing esophageal varices: 1-versus 2-week intervals. *Hepatogastroenterology*, **36** 337–340
27. Westaby, D., Melia, W.M., MacDougall, B.R.D., Hegarty, J.E. and Williams, R. (1984) Injection sclerotherapy for oesophageal varices: A prospective randomised trial of different treatment schedules. *Gut*, **25** 129–132
28. Sarin, S.K., Sachdev, G., Nanda, R.M., Batra, S.K. and Anand, B.S. (1986) Comparison of the two time schedules for endoscopic sclerotherapy: a prospective randomised controlled study. *Gut*, **27** 710–713
29. Warren, W.D., Henderson, J.M., Millikan, W.J., Galambos, J.T., Scott Brooks, W., Riepe, S.P., Salam, A.A. and Kutner, M.H. (1986) Distal splenorenal shunt versus endoscopic sclerotherapy for long-term management of variceal bleeding. Preliminary report of a prospective randomized trial. *Annals of Surgery*, **203** 454–462
30. Cello, J.P. Grendell, J.H., Crass, R.A., Weber, T.E. and Trunkey, D.D. (1987) Endoscopic sclerotherapy versus portocaval shunt in patients with severe cirrhosis and acute variceal haemorrhage – long-term follow-up. *New England Journal of Medicine*, **316** 11–15

